# Anonymisierung von Feldinhalten in hausärztlichen Behandlungsdaten – Exemplarische Untersuchung an zwei Forschungsdatensätzen

**DOI:** 10.1055/a-2624-0084

**Published:** 2025-07-14

**Authors:** Johannes Hauswaldt, Roland Groh, Knut Kaulke, Falk Schlegelmilch, Alireza Zarei, Eva Hummers

**Affiliations:** 1 Institut für Allgemeinmedizin, Universitatsmedizin Göttingen, Göttingen, Germany; 2 AG "Anwendungs- und Informationssysteme", Gesellschaft für Wissenschaftliche Datenverarbeitung mbH Göttingen, Göttingen, Germany; 3 Data protection, Technologie- und Methodenplattform für die vernetzte medizinische Forschung (TMF), Berlin, Germany; 4 Department of General Practice, University Medical Center Göttingen, Göttingen, Germany

**Keywords:** Datenschutzfolgenabschätzung, Routinedaten, Praxisverwaltungssystem, Identifikator, Text mining, Natural language processing, data protection impact assessment, routine data, practice management system, identifier, text mining, natural language processing

## Abstract

**Einleitung:**

Daten eines Datensatzes können nur dann als „anonym“ bezeichnet werden, wenn sie keinesfalls und auch nicht nachträglich, auch nicht durch Verknüpfung mit weiteren Informationen, auf eine Person bezogen werden können. Potentiell identifizierende Feldinhalte (PIF) verhindern die „faktische Anonymisierung“ eines wissenschaftlich genutzten Sekundärdatensatzes. An zwei Quelldatensätzen aus hausärztliche Praxisdaten wurde exemplarisch untersucht, ob und inwieweit schrittweises und systematisches Erkennen von PIF möglich ist.

**Methodik:**

Von 14.285 bzw. 100 Patient*innen wurden aus hausärztlichen Praxisverwaltungssystemen Routinedaten mit 40 Variablen (Parametern, Feldern) in 5.918.321 bzw. 363.084 Datenzeilen exportiert und auf den vier Ebenen ihrer Feldkennungen, deren Kombinationen, ihrer Feldinhalte sowie des gesamten Datensatzes untersucht. Häufigkeiten von Feldkennungen wurden in elf semantischen Gruppen sowie nach Feldtypen geordnet. Die Gefahr einer Re-Identifizierung insbesondere bei Kombination von Feldkennungen wurde aus hausärztlicher Expertise beurteilt. In schrittweise iterativem Vorgehen untersuchten wir auf PIF bei Freitexteinträgen und maskierten Treffer für die nachfolgenden Durchgänge. Der abschließende Quotient aus Anzahl re-identifizierender und Gesamtzahl aller Datenzeilen bildete den Wahrscheinlichkeitsschätzer. Zusätzlich wurden die Daten in Gänze mittels einer Open-Source-Software zur Anonymisierung sensibler personenbezogener Daten beurteilt. Zuletzt erfolgte eine Ergebnisbewertung im Sinne einer Datenschutz-Folgenabschätzung nach Art. 35 der DSGVO bezüglich Schwere eines möglichen Schadens und seiner Eintrittswahrscheinlichkeit.

**Ergebnisse:**

Unter den zur wissenschaftlichen Sekundärnutzung ausgewählten 40 Parametern beurteilten wir insbesondere Freitextfelder wie „Dauerbemerkungen“, „Aktuelle Diagnose“, „Medikament“ und „Befund“ als re-identifizierend. Akribische Vorabauswahl und Datensparsamkeit, *privacy by design* im Verarbeitungsprozess sowie die hier beschrieben de-identifizierende Maßnahmen verringerten dieses Risiko erheblich, konnten jedoch einen „faktisch anonymen“ Sekundärdatensatz insgesamt nicht erreichen.

**Schlussfolgerung:**

Erkennen und Bewerten von PIF sind Voraussetzung für de-identifizierende Maßnahmen, sind jedoch mit vertretbarem Aufwand immer nur unvollständig erfolgreich. Eine semantische Strukturierung der Daten ist erstrebenswert, hilft jedoch der Möglichkeit einer Re-Identifizierung durch Fehleingaben nicht ab.

## Abstract (en)

**Introduction:**

A dataset can be called “anonymous” only if its content cannot be related to a person, not by any means and not even *ex post* or by combination with other information. Free text entries highly impede “factual anonymization” for secondary research. Using two source datasets from GPs’ electronic medical records (EMR), we aimed at de-identification in an iterative and systematic search for potentially identifying field content (PIF).

**Methods:**

EMR data of 14,285 to 100 GP patients with 40 variables (parameters, fields) in 5,918,321 resp. 363,084 data lines were analyzed at four levels: field labels, their combination, field content, dataset as a whole. Field labels were arranged into eleven semantic groups according to field type, their frequencies examined and their combination evaluated by GP experts rating the re-identification risk. Iteratively we searched for free text PIFs and masked them for the subsequent steps. The ratio of PIF data lines’ number over total number yielded final probability estimators. In addition, we processed a whole dataset using ARX open source software for anonymizing sensitive personal data. Results were evaluated in a data protection impact assessment according to article 35 GDPR, with respect to the severity of privacy breach and to its estimated probability.

**Results:**

We found a high risk of re-identification with free text entries into “history”, “current diagnosis”, “medication” and “findings” even after repeated algorithmic text-mining and natural language processing. Scrupulous pre-selection of variables, data parsimony, privacy by design in data processing and measures described here may reduce the risk considerably, but will not result in a “factually anonymized” research dataset.

**Conclusion:**

To identify and assess re-identifying field content is mandatory for privacy protection but anonymization can be reached only partly by reasonable efforts. Semantic structuring of data is pre-conditional but does not help with erroneous entries.

## Einleitung

Daten eines Datensatzes können nur dann als „anonym“ bezeichnet werden, wenn sie weder durch den Verantwortlichen selbst noch durch Dritte, auch nicht durch Verknüpfung mit weiteren Informationen, auf eine Person bezogen werden können 1 2 . In dieser Untersuchung werden vorgefundene alphanumerische Einträge (Variablenausprägungen), also Texte oder Zahlenwerte, in einem Feld (Variable, Parameter) eines strukturierten Datensatz als potentiell identifizierende Feldinhalte (PIF) kategorisiert, die, allein oder in Kombination mit Einträgen in anderen Feldern, nachfolgend die Re-Identifikation eines Betroffenen möglich machen; PIF verhindern die „faktische Anonymisierung“ eines wissenschaftlich genutzten Sekundärdatensatzes.

Daten können für einen Verantwortlichen anonym sein können, wenn dieser nicht durch Verknüpfung mit Informationen, die ihm zur Verfügung stehen, einen Personenbezug herstellen kann. Das Konzept des relativen Personenbezugs wurde mittlerweile mehrfach vom Europäischen Gerichtshof (EuGH) bestätigt, es besagt, dass die Anonymität für diesen spezifischen Verantwortlichen bestehen bleibt, selbst wenn andere Verantwortliche eine Zuordnung herstellen könnten 3 4 .

Behandlungsdaten der Hausarztpraxis haben ein großes Nutzungspotenzial für die allgemeinmedizinische Forschung und darüber hinaus für die Versorgungs- und Gesundheitssystemforschung, weil sie unter Realbedingungen aus Patientenbehandlungen als primärem Zweck erwachsen und diese longitudinal, über lange Zeiträume abbilden 5 . Allermeist haben diese besonders schützenswerten Daten zunächst einen Personen- bzw. Patientenbezug, so dass deren Nutzung für die Forschung (Sekundärdatennutzung) strengen Anforderungen an Patienteneinwilligung und Datenschutz genügen muss: personenbezogene Daten dürfen nicht genutzt werden, es sei denn, es läge ein Erlaubnistatbestand vor, in der Regel eine informierte Einwilligung des Betroffenen oder eine gesetzliche Regelung. Datennutzung für Forschungszwecke bedeutet eine Zweckänderung gegenüber dem ursprünglichen Zweck der Datennutzung zur Patientenbehandlung und bedarf deshalb der erneuten Einwilligung durch den Betroffenen. Dass die sekundäre Nutzung von Behandlungsdaten aus hausärztlichen Praxisverwaltungssystemen technisch und organisatorisch rechtskonform machbar ist, konnte in den DFG-geförderten Projekten RADAR (2016 bis 2020) und RADARplus gezeigt werden 6 7 . Jedoch ist eine Nutzung dieser Daten, die derart auf individueller informierter Einwilligung basiert, für die Forschung zeit- und ressourcenaufwändig und kann daher nur kleine Datenmengen für die Forschung liefern.

Vom deutschen Gesetzgeber wurde das Potenzial in Gesundheitsdaten erkannt, wenn diese über den originären Behandlungszweck hinaus auch sekundär für die gemeinwohlorientierte Forschung genutzt werden. Das „Gesetz zur verbesserten Nutzung von Gesundheitsdaten (GDNG)“ 8 regelt die Nutzung von Gesundheitsdaten für gemeinwohlorientierte Forschung und soll zu deren vereinfachter Forschungsnutzung beitragen. Eine dieser Vereinfachungen könnte durch einwilligungsfreie Datennutzung nach den Regelungen in § 6 des GDNG entstehen, wenn in datenverarbeitenden Gesundheitseinrichtungen die Gesundheitsdaten bereits in der Arztpraxis durch befugte Personen anonymisiert werden, um sie dann für Forschungszwecke zu übermitteln. Das konkrete Vorgehen nach § 6 GDNDG in der Arztpraxis bedarf noch der Ausgestaltung, eine Anonymisierung der Gesundheitsdaten jedoch ist dabei essenzielle Voraussetzung, die zum frühestmöglichen Zeitpunkt eingesetzt werden sollte.

An zwei Quelldatensätzen aus hausärztlichen Praxisdaten wurde exemplarisch untersucht, ob und inwieweit schrittweises und systematisches Erkennen von PIF möglich ist. Diese Untersuchung zielte darauf ab, die Herausforderungen und Grenzen der faktischen Anonymisierung von Primärdaten aus Sicht verschiedener Verantwortlicher zu untersuchen.

## Methodik

Aus einem vorliegenden vollständigen Datenexport von Routinedaten im Praxisverwaltungssystem einer Hausarztpraxis mittels der Behandlungsdaten-Transfer Schnittstelle (BDT) für den Zeitraum 01.01.1994 bis 31.12.2017 von N=14.285 Patient*innen wurden die Inhalte von 40 im RADAR Projekt 6 7 beschriebenen BDT-Variablen (Parametern, Feldern) extrahiert und ergaben den ersten Quelldatensatz ( *source data set 1* , SDS1) mit 5.918.321 Datenzeilen. In gleicher Weise wurden Routinedaten aus sieben Hausarztpraxen, 01.01.2012 bis 31.03.2019, von N=100 Patient*innen mit oraler Antikoagulation als Inhalte von 40 BDT-Variablen gewonnen und bildeten den zweiten Quelldatensatz ( *source data set 2* , SDS2) mit 363.084 Datenzeilen. Bei diesen Exporten standen entsprechend dem Datenschutz- und IT-Sicherheitskonzept der RADAR-Projekte 6 7 den untersuchenden Wissenschaftlern allein medizinische Daten (MDAT) zur Verfügung, nicht jedoch die identifizierenden Daten (IDAT), die bei der Vertrauensstelle blieben. Direkte Identifikatoren waren also für die Untersucher von vornherein ausgeschlossen ( *privacy by design* ).

Diese beiden strukturierten Quelldatensätze untersuchten wir getrennt und auf den vier Ebenen (a) ihrer Feldkennungen (Variablenbezeichnungen, Attribute), (b) derer Kombinationen, (c) ihrer Feldinhalte (Ausprägungen, Werte), sowie (d) des gesamten Datensatzes. Für (a) und (b) betrachteten wir insbesondere den Feldtyp, die absoluten und relativen Häufigkeiten ihres Auftretens, ihre Einordnung in elf semantische Gruppen (Diagnosen, Medikation, Laborergebnisse, Befunde, Therapien, weitere Prozeduren, Zeit- und Datumsdaten, Stamm- und Dauerdaten des Patienten, Kenndaten der Praxis, Kostenträger, Abrechnung) 9 , und beurteilten jeweils das Risiko einer Re-Identifizierung aus hausärztlicher Expertise. Bei (c) untersuchten wir in iterativem Vorgehen die Feldinhalte auf potentiell identifizierende Informationen, insbesondere in Feldern mit Freitexteinträgen. Hierbei wurden sowohl direkte Identifikatoren (wie Name, Adresse) als auch Quasi-Identifikatoren (wie Alter, Geschlecht) bewertet. Gefundene potentiell identifizierende Feldinhalte (PIF) wurden für die nachfolgenden Durchgänge de-identifiziert (maskiert). Dabei nutzten wir bei jeder Schrittwiederholung zum einen die Anwendungssoftware *TextCrawler* in der Version 3.1.2 10 und anschließend zum anderen Instrumente des *Natural Language Processing* (NLP), zum Beispiel 11 .

In einem BDT-Datensatz entspricht eine Datenzeile einem Feld (Variable, Parameter), die den jeweiligen Feldinhalt (Variablenausprägung, Wert) enthält. Als Schätzer für die Wahrscheinlichkeit, in einem derartig abgeschlossenen und strukturierten Datensatz PIF zu finden, berechneten wir abschließend den Wahrscheinlichkeitsschätzer



wobei n die Anzahl PIF-enthaltender und N die Gesamtzahl der Datenzeilen eines Datensatzes ist. Wir haben diesen einfachen Schätzer eingeführt, weil über eine akzeptierte Obergrenze für die Wahrscheinlichkeit, in einem Datensatz re-identifizierende Informationen zu finden, ein allgemeiner Konsens nicht besteht.

Zusätzlich wurde SDS1 als ganzer Datensatz mittels ARX, einer Open-Source-Software zur Anonymisierung sensibler personenbezogener Daten, die eine Vielzahl von Datenschutz- und Risikomodellen unterstützt 12 , von uns untersucht und beurteilt. Wir nutzten hierfür die Version ARX-3.9.1.

Zuletzt nahmen wir eine Ergebnisbewertung im Sinne einer Datenschutz-Folgenabschätzung nach Art. 35 der DSGVO bezüglich Schwere eines möglichen Schadens und seiner Eintrittswahrscheinlichkeit vor.

## Ergebnisse

Als problematisch, weil möglicherweise PIF enthaltend, sind unter den zur wissenschaftlichen Sekundärnutzung ausgewählten 40 BDT-Feldkennungen eines Routinedatensatzes aus hausärztlichen Praxisverwaltungssystemen insbesondere die Freitextfelder „Dauerbemerkungen“ (FK 3656), „Aktuelle Diagnose“ (FK 6205), „Medikament auf Rezept“ (FK 6210), „Befund“ (FK 6220) und „Röntgenbefund“ (FK 6225) zu beurteilen. Es finden sich unmittelbar personenidentifizierende Einträge oder auch in semantischer Hinsicht Fehleinträge, die als Identifikatoren genutzt werden können. Das Feld „PLZ Praxisort“ (FK 0204) halten wir in den medizinischen Daten (MDAT) für entbehrlich, weil es bereits in den Identitätsdaten (IDAT) enthalten ist, die für die Treuhandstelle separiert wurden, und haben es daher entfernt. Eine Treuhandstelle agiert in diesem Kontext zwischen den Forschungsdatengebern und der Nachfrageseite von Forschungsdaten als unabhängige und neutrale Vertrauensinstanz. Sie unterliegt idealerweise einer besonderen Geheimhaltungspflicht und kann beispielsweise ein Notar oder ein externer Arzt sein 13 . Während wir das „Geschlecht des Patienten“ (FK 3110) unverändert ließen, reduzierten wir im RADAR Projekt das „Geburtsdatum des Patienten“ (FK 3103) auf sein Geburtsjahr.

Vor allem in den drei Feldern „Dauerbemerkungen“, „Medikament“ und „Befund“, siehe oben, fanden wir als Freitexteinträge in den beiden Sekundärdatensätzen Kurzmitteilungen oder fälschlicherweise abgelegte Laborbefunde, Eigennamen, Berufs-, Funktions- oder Verwandschaftsbezeichnungen, Diagnosen, das Datum des Versterbens oder Telefonnummern. Listenbasiert oder für Telefonnummern algorithmisch durchsuchten wir wiederholt SDS1 und SDS2 mittels der Software *TextCrawler* und maskierten jeweils identifizierende Treffer für nachfolgende Durchgänge. Die Resultate untersuchten wir dann wiederum mittels *Natural Language Processing* (NLP) mit Filtern für „Ort“ und „Person“. Die abschließenden Ergebnisse für Gesamtzahl sowie für die bei nachfolgender „händischer Kontrolle“ richtig-positiven der jeweiligen Treffer, dazu beobachtete Feldeinträge, die falsch-negativ waren und deshalb nicht als Treffer bezeichnet wurden, sind in Tab. 1 zu finden, ebenso die daraus berechneten Wahrscheinlichkeitsschätzer.

**Table:** **Tab. 1** Anzahl und geschätzte Wahrscheinlichkeit gefundener Treffer (gesamt, richtig-positiv, falsch-negativ) mittels TextCrawler® und *Natural Language Processing (NLP)* , siehe Text

	Treffer	Sekundärdatensatz 1		Sekundärdatensatz 2	
		Anzahl Datenzeilen	*Wahrscheinlich-keit (Schätzer)*	Anzahl Datenzeilen	*Wahrscheinlich-keit (Schätzer)*
		**n**	***p̂***	**n**	***p̂***
		5 918 321	*1,00000000*	340 082	*1,00000000*
**Arztnamen**	Gesamt	104.025	*0,01757678*	5499	*0,01616963*
	Richtig-positiv	68	*0,00001149*	176	*0,00051752*
	Falsch-negativ	1	*0,00000017*	1	*0,00000294*
**Namen**	Gesamt	31 140	*0,00526163*	2625	*0,00771873*
	Richtig-positiv	254	*0,00004292*	76	*0,00022348*
**Städtenamen**	Gesamt	15 227	*0,00257286*	234	*0,00068807*
	Richtig-positiv	34	*0,00000574*	2	*0,00000588*
**Telefonnummern und Postleitzahlen**	Gesamt	2553	*0,00043137*	9	*0,00002646*
	Telefonnnumern, richtig-positiv	0		1	*0,00000294*
	Telefonnummern, falsch-negativ	1	*0,00000017*	0	
	PLZ, richtig-positiv	0		31	*0,00009115*
***NLP, location filtered***	Gesamt	7639	*0,00129074*	46	*0,00001353*
	Richtig-positiv	3	*0,00000051*	26	*0.00000765*
	Falsch-negativ	1	*0,00000017*		
***NLP, person filtered***	Gesamt	77 652	*0,01312061*	1825	*0.00536635*
	Richtig-positiv	433	*0,00007316*	51	*0.00001499*

In den mittels *TextCrawler* untersuchten Datensätzen SDS1 und SDS2 wurden Arztnamen in 68 bzw. 176 Datenzeilen als richtig-positive Treffer gefunden, für die daraus errechneten Wahrscheinlichkeitsschätzer siehe Tab. 1 . Zusätzlich wurden Namen in 254 bzw. 76 Fällen korrekt gefunden, Städtenamen in 34 bzw. 2 Fällen, Telefonnummern nicht bzw. einmal. Die Zahl der durch die Untersuchungsinstrumente angezeigten Treffer lagen zwar um ein Vielfaches höher, siehe Tab. 1 , jedoch erwiesen sich diese bei einer „händischen“ Kontrolle, also einem vollständigen Durchmustern der Treffer durch einen menschlichen Untersucher, allermeist als falsch-positive Treffer. Allerdings fanden sich bei diesen manuellen Kontrollen auch mehrfach von *TextCrawler* nicht erkannte Einträge, die in entsprechender Weise als falsch-negative Ergebnisse zu bewerten sind.

Die derart gewonnenen, in mehrfachen Durchläufen mittels *TextCrawler* de-identifizierten Datensätze SDS1 und SDS2 wurden erneut mit Instrumenten des *Natural Language Processing* (NLP) untersucht. Auch hier wurden für die Suchfunktion „ *location filtered* “ 3 bzw. 26 richtig-positive, für „ *person-filtered* “ noch weitere 433 bzw. 51 richtig-positive Treffer gefunden. Die Anzahl der angezeigten Treffer, die sich bei Nachkontrolle als falsch-positiv erwiesen, erwies sich ebenfalls als hoch. Und auch hier fanden wir händisch einen Treffer, der nicht angezeigt worden war, also als falsch-negativ eingeordnet werden muss, siehe Tab. 1 .

Das Untersuchungsinstrument ARX wird auf abgeschlossene strukturierte Datensätze *in toto* angewendet. Es unterscheidet drei Modelle re-identifizierender Angriffe ( *Prosecutor attacker model, Journalist attacker model, Marketer attacker model* ). Wir haben beispielhaft SDS1 als den größeren der beiden vorliegenden Datensätze mit 5.918.321 Datenzeilen untersucht. Die Ergebnisse einer Risikoanalyse mittels ARX finden sich in Tab. 2 .

**Table:** **Tab. 2** Risikoanalyse des ersten Quelldatensatzes (SDS1) mittels ARX, siehe Text

*Measure*	*Value [%]*
*Lowest prosecutor risk*	*0.0002%*
*Records affected by lowest risk*	*8.62502%*
*Average prosecutor risk*	*5.19889%*
*Highest prosecutor risk*	*100%*
*Records affected by highest risk*	*2.88005%*
*Estimated prosecutor risk*	*100%*
*Estimated journalist risk*	*100%*
*Estimated marketer risk*	*5.19889%*
*Sample uniques*	*2.88005%*
*Population uniques*	*0.53364%*

Lediglich für die schwächste Form eines Re-Identifizierungsangriffs ( *marketer attack* ), der sich allein auf den vorgefundenen Datensatz stützt, wird ein Identifizierungsrisiko von 5,2% angegeben: 5 von 100 Datensätzen dieser Art und Form ermöglichen eine Re-Identifizierung eines Betroffenen. Für die beiden anderen Angriffsmodelle *(journalist attack* und *prosecutor attack* ) wird ein Risiko von 100% angenommen, d. h. in der *worst case* Annahme dieser beiden Angriffsformen durch ARX wird durch Zusammenführen und Verknüpfen der Informationen eines solchen Datensatzes mit sonstigen, öffentlich aufzufindenden oder gar mit auf jegliche legale Art recherchierbaren personenbezogenen Daten in jedem Fall mindestens eine Re-Identifizierung möglich.

**Abb. 1:**
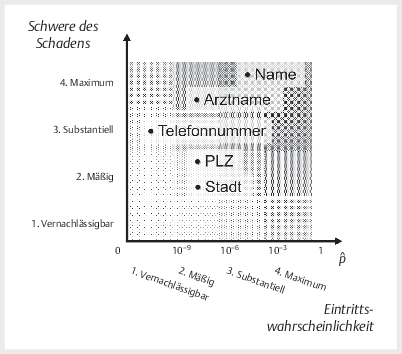
Datenschutzfolgenabschätzung.

Zum Schluss führten wir eine Bewertung unserer Ergebnisse im Sinne einer Datenschutzfolgenabschätzung entsprechend Art. 35 der DSGVO durch. Hierfür stellten wir die Schwere eines möglichen Schadens mit seiner Eintrittswahrscheinlichkeit zusammen. Als Schaden betrachteten wir re-identifizierende Hinweise auf Personen, in unserer Untersuchung „Namen“, „Arztnamen“, Telefonnummern“, „Postleitzahlen“ und „Orte/Stadt“, die wir entsprechend unseren Ergebnissen in ein übliches semi-quantitatives Koordinatensystem aus Schwere des Schadens und seiner Eintrittswahrscheinlichkeit einordneten, siehe Abb. 1 .

Besonders kritisch ist regelmäßig die Kombination mehrerer Informationen zu bewerten, da möglicherweise erst durch die Verknüpfung mehrerer Datenpunkte ein Schaden entsteht. So stellt beispielsweise das alleinige Bekanntwerden einer Postleitzahl in der Regel keinen erheblichen Schaden dar, die Kombination von möglicherweise patientenidentifizierenden Informationen mit einem Arztnamen jedoch sehr wohl. Bereits früher konnten wir zeigen, dass beispielsweise das Duplet, gebildet aus „Altersdekade“ und „Geschlecht“ eines Patienten, in dem Datensatz einer Hausarztpraxis, 2012 bis 2016, eine k-Anonymität von k=10 mehrfach unterschreitet und deshalb bereits diese Information nicht als „faktisch anonym“ anzusehen ist (Ergebnisse nicht veröffentlicht). Weitere gängige Kombinationen von personenbezogenen Informationen, beispielsweise Triplets aus Patientenidentifikator und Kalenderdatum mit Diagnosen oder Medikation, sind ebenfalls als re-identifizierend einzuschätzen 9 . Letztlich können die 11 semantischen Gruppen 9 bei ihrer gemeinsamen wissenschaftlichen Betrachtung keinesfalls als „faktisch anonym“ angesehen werden.

## Diskussion

Während der sekundären Nutzung von Routinedaten aus hausärztlichen Praxisverwaltungssystemen für wissenschaftliche Fragestellungen sind umfassende Maßnahmen zum Datenschutz unabdingbar.

Von Beginn des RADAR Projekts und mit der Entwicklung seines Datenschutz- und IT-Sicherheitskonzepts 2014 bis 2017 wurden *Privacy by design* , beispielsweise durch Trennung von MDAT und IDAT, und Datenminimierung, etwa durch Beschränkung auf 40 Feldkennungen für die sekundäre Datennutzung, eingeführt. Weitere Einsparungen folgten, beispielsweise indem das Geburtsdatum von Patient*innen durch ihr Geburtsjahr ersetzt wurde. Letztlich konnte durch diese und weitere technische und organisatorische Maßnahmen die rechtskonforme Nutzung pseudonymer, also einwilligungsbasierter Routinedaten für Forschungszwecke als machbar nachgewiesen werden.

Unsere hier berichteten nachfolgenden Anstrengungen im Projekt RADARplus, PIF mittels verschiedener algorithmischer Verfahren aus den zwei vorliegenden Datensätze in vollem Umfang zu entfernen, waren nicht gänzlich erfolgreich: neben einer großen Zahl von Treffern aus automatisierter Suche, die sich bei „händischer“ Kontrolle als falsch-positiv, also nicht als personenidentifizierend erwiesen (beispielsweise Eigennamen von Pharmafirmen), fanden sich in beiden Datensätzen nach Abschluss des Textminings immer noch richtig-positive Treffer sowohl beim nachgeschalteten Durchsuchen mittels Natural Language Processing (NLP) als auch zum Schluss bei „händischer“ Endkontrolle. Noch bedenklicher erscheint uns, dass auch nach Abschluss aller Verfahren durch abschließendes menschliches Durchsuchen in beiden Datensätzen noch vereinzelt falsch-negative Treffer, also zuvor nicht erkannte PIF, gefunden wurden (siehe Tab. 1 ). Zwar sind die absoluten Anzahlen dieser gesuchten, richtig-positiv und falsch-negativ, Treffer klein und damit die zugehörigen Wahrscheinlichkeitsschätzer im Bereich zwischen 10 ^-5^ und 10 ^-7^ pro Datenzeile, dennoch müssen wir festhalten, dass eine Datensatzaufbereitung, die zur oben definierten faktischen Anonymität führt, nicht gelang. Mit vertretbarem Aufwand können letztlich PIF in einem abgeschlossenen SDS immer nur unvollständig erkannt werden. Erkennen und Bewerten von PIF sind jedoch Voraussetzung für de-identifizierende Maßnahmen.

Das weitreichende Risiko einer Re-Identifizierung durch Kombinationen von Informationen und Datenpunkten − informationstheoretisch betrachtet, letztlich immer eine Verringerung von Entropie − konnten wir hier nicht quantifizieren, sondern lediglich andeuten.

„Die Pseudonymisierung – Namensverschleierung – soll verhindern, dass konkrete Personen aus den Datensätzen re-identifiziert werden können. Sehr detaillierte Datensätze sind jedoch schwer sinnvoll durch Namensersetzung zu maskieren, weil Menschen im Detail doch sehr individuelle Biographien haben. Zudem sind gerade die zusammenhängenden individuellen Krankengeschichten und Sachhergänge für die medizinische Forschung relevant – dies gilt umso mehr bei seltenen Diagnosen und Krankheiten …“ 14 . Noch mehr gilt dies für alle Ansätze der Anonymisierung 15 .

Untersuchungen zu PIF müssen immer an einem konkreten, abgeschlossen vorliegenden SDS durchgeführt werden. Sie setzen fach- und sachspezifische Kenntnisse über Entstehung und Rahmenbedingungen der Rohdaten in Hausarztpraxen sowie Metainformationen über die Primärdaten voraus. Eine semantische Strukturierung der Daten, etwa unter SNOMED CT, ist erstrebenswert, hilft jedoch nicht ab, wenn PIF durch semantische Fehleingaben erwachsen.

Die Problematik einer Anonymisierung von medizinischen Individualdaten ist seit langem Gegenstand der wissenschaftlichen Diskussion, siehe beispielhaft den Beitrag von Prasser und Sariyar, 2017, in 16 . Noch mehr als bei der Pseudonymisierung von Daten wird diese − weil regelmäßig ohne Einwilligung der Betroffenen durchgeführt − von der dilemmatischen Spannung zwischen dem grundlegenden Recht des Betroffenen auf informationelle Selbstbestimmung, mit dem Schutz seiner sensiblen Gesundheitsdaten vor Re-Identifizierung, einerseits und dem individual- und gemeinwohlorientierten Interesse an der sekundären Aufarbeitung und wissenschaftlichen Nutzung dieser Gesundheitsdaten andrerseits bestimmt. Wie auch unsere Untersuchung hier erneut bestätigt, zeigte sich, dass Versuche der De-Identifizierung auf der Ebene eines Datensatzes allein nicht ausreichend sind: das Entfernen von direkten Identifikatoren, Datensparsamkeit und Verzicht auf Datenfelder, nachträgliche Maßnahmen zur Maskierung, Vergröberung durch Zusammenfassung oder Anbringen zufallsgebundener Unschärfe etc. reichen nicht aus, um eine dann noch sinnvolle Anonymisierung verlässlich zu erreichen.

Es bedarf vielmehr darüber hinaus weiterer Maßnahmen und dauerhaft-umfassender Vorkehrungen, um das genannte Dilemma ausreichend zu versöhnen, siehe ebenfalls 16 , insbesondere einer geschützten Forschungsdateninfrastruktur und einer jeweils *ex ante* errichteten Architektur für die sekundäre wissenschaftliche Nutzung von *Real-world* -Behandlungsdaten, mit „ *privacy by design* “, Transparenz durch Zugangskontroll- und Auswahlgremien sowie vertraglicher Selbstverpflichtung aller Verantwortlichen.

Eine wesentliche und wirksame Komponente in einer derartigen Architektur ist die frühzeitig getrennte Gewinnung von identifizierenden (IDAT) und medizinischen Daten (MDAT) sowie deren separate Verarbeitung unter Einschaltung einer unabhängigen Vertrauensstelle ( *trusted third party* , TTP), siehe etwa 6 7 . Bei Druschke et al. 17 findet sich ein gutes Beispiel für ein vorab wohlüberlegtes Studiendesign für die Verknüpfung ( *data linkage* ) mehrerer Datenquellen, mit strikt getrennten Akteuren in deren Rollen, mehreren unterschiedlichen Datenhaltern im Verarbeitungsprozess sowie ebenfalls einer Vertrauensstelle.

Specht-Riemenschneider und Heineking 18 legen beispielhaft eine aktuelle, umfassende und klare Übersicht zur Datennutzung in sicheren Verarbeitungsumgebungen vor. Auch sie verweisen auf das genannte Dilemma, deklinieren datenschutzrechtliche Anforderungen, Rechtmäßigkeit der Datenverarbeitung, Datenerhebung für Behandlungs- und für wissenschaftliche Forschungszwecke, rechtliche Folgen einer Zweckänderung für die erlaubte Weiterverarbeitung einschließlich einer Kompatibilitätsprüfung dabei, und diskutieren Interessen am Unterbleiben und an der Durchführung der Verarbeitung. Sie führen zusätzliche technische und organisatorische Maßnahmen zur Senkung der Risiken der Verarbeitung an, u. a. namentlich die Protokollierung der Übermittlungstätigkeiten, Gewähr der Einsicht für die zuständigen Aufsichtsbehörden sowie Nachweis der Erfüllung vertraglicher Verpflichtungen des Empfängers übermittelter Daten zur Einhaltung des Verarbeitungszwecks. Ebenfalls plädieren sie für den Einsatz von Datentreuhändern zur wesentlichen Reduzierung der Verarbeitungsrisiken und fordern vom Gesetzgeber, Rechtssicherheit dabei für die wissenschaftlich Datenverarbeitenden durch geeignete Rahmensetzung zu gewährleisten. Das GDNG leistet dies in Teilen, erforderlich ist jedoch ein umfassendes Forschungsdatengesetz mit exkulpierender Wirkung für die im Gemeinwohl tätigen Wissenschaftler.

## Fundref Information

Deutsche Forschungsgemeinschaft — http://dx.doi.org/10.13039/501100001659; HU 1587/2-2
